# Fatigue Performance of Q500qENH Weathering Steel Welded Joints at Low Temperature

**DOI:** 10.3390/ma18194515

**Published:** 2025-09-28

**Authors:** Lei Kang, Xuanming Shi, Tao Lan, Xiaowei Zhang, Chen Xue, Xiaopeng Wang, Zhengfei Hu, Qinyuan Liu

**Affiliations:** 1 School of Materials Science and Engineering, Tongji University, Shanghai 201804, China; leikang45@163.com; 2 CSSC International Engineering Co., Ltd., Beijing 100121, China; xuanming_shi_tj@163.com (X.S.); wilsonzw2019@outlook.com (X.Z.); xuechen_2020@163.com (C.X.); l547790690@163.com (Q.L.); 3 College of Civil Engineering, Tongji University, Shanghai 200092, China; xiaopengwang@tongji.edu.cn; 4 School of Civil Engineering, Xi’an University of Architecture and Technology, Xi’an 710055, China

**Keywords:** weathering steel, welded joints, low temperature, fatigue crack propagation, fatigue life

## Abstract

A systematic study was conducted on the fatigue performance of Q500qENH weathering steel welded joints under low-temperature conditions of −40 °C in this paper. Low-temperature fatigue tests were conducted on V-groove butt joints and cross-shaped welded joints and S-N curves with a 95% reliability level were obtained. A comparative analysis with the Eurocode 3 reveals that low-temperature conditions lead to a regular increase in the design fatigue strength for both types of welded joints. Fracture surface morphology was examined using scanning electron microscopy, and combined with fracture characteristic analysis, the fatigue fracture mechanisms of welded joints under low-temperature conditions were elucidated. Based on linear elastic fracture mechanics theory, a numerical simulation approach was employed to investigate the fatigue crack propagation behavior of welded joints. The results indicate that introducing an elliptical surface initial crack with a semi-major axis length of 0.4 mm in the model effectively predicts the fatigue life and crack growth patterns of both joint types. A parametric analysis was conducted on key influencing factors, including the initial crack size, initial crack location, and initial crack angle. The results reveal that these factors exert varying degrees of influence on the fatigue life and crack propagation paths of welded joints. Among them, the position of the initial crack along the length direction of the fillet weld has the most significant impact on the fatigue life of cross-shaped welded joints.

## 1. Introduction

Weathering steel, also known as atmospheric corrosion-resistant steel, is a type of low-alloy steel produced by adding appropriate amounts of alloying elements such as copper, nickel and chrome to conventional steel. Compared to ordinary steel, it exhibits superior resistance to atmospheric corrosion and enhanced low-temperature impact toughness, along with excellent properties such as high strength, toughness, ductility, and corrosion resistance. These characteristics make it well-suited for the low-temperature environments of plateau regions. The use of weathering steel in bridge construction helps reduce the lifecycle costs of steel structures. Consequently, in recent years, weathering steel has been widely adopted in bridge engineering projects both domestically and internationally.

In cold regions, large-scale infrastructure such as bridges is subjected to prolonged exposure to low-temperature environments during service, along with complex and variable loading conditions. Consequently, numerous safety concerns have emerged, among which fatigue-induced structural failure has become one of the most critical challenges in infrastructure. Many researchers have investigated the low-temperature fatigue behavior of steels: Li et al. [1] studied the fatigue performance of fillet-welded joints in Q420C steel under low temperatures and found that fatigue failures consistently occurred at the weld toe. Jia [2] conducted fatigue tests on base metal and butt-welded joints of Q345qD steel at various temperatures (0 °C, −20 °C, −40 °C, −60 °C), demonstrating that fatigue strength improved with decreasing temperature for both materials. Wu et al. [3] performed high-cycle fatigue tests on imported EA4T axle steel at both room temperature and low temperature (−40 °C), observing a 28.7% increase in the fatigue limit under cryogenic conditions compared to ambient temperature. However, most studies have focused on conventional steels, whereas systematic research on the low-temperature fatigue performance of high-strength weathering steel remains limited.

In fatigue design, the S-N curve based on nominal stress and hot spot stress [4,5,6,7,8,9,10], along with the Palmgren–Miner accumulative damage rule [11], is commonly used to assess fatigue damage. However, when fatigue details are not covered by design codes or when the structure is subjected to multiaxial stress states, the S-N curve-based approach may no longer be applicable [12]. The Notch stress method overcomes the difficulties associated with determining nominal stresses in welded structures and defining structural stresses at weld roots. It provides a more realistic representation of the local stress–strain state in welded joints, significantly improving the accuracy of fatigue life prediction [13,14,15,16]. Nevertheless, the notch stress model is relatively complex to construct, involves large-scale computations, and requires extensive time to establish S-N curves when the loading history is unknown [17]. Meanwhile, very recent entropy-based fatigue models have emerged. These methods quantify damage accumulation through entropy generation, offering potential advantages for complex structures and variable loads. However, challenges related to material, load dependency and model complexity remain [18,19,20]. Because of the limitations of the aforementioned methods, fracture mechanics method [21,22,23] can also be adopted for fatigue assessment, which focus on the microscopic mechanisms of crack initiation and propagation. Since welding inevitably introduces initial cracks and defects in steel bridges [24], and most steel bridges remain in a fully elastic state under vehicular loads, linear elastic fracture mechanics theory (LEFM) is particularly well-suited for fatigue evaluation of steel bridges.

This study examines the corrosion and fatigue performance of Q500qENH weathering steel under extreme conditions, filling a research gap compared to conventional steel studies. It uniquely combines S-N curve analysis with fracture mechanics to investigate the impact of initial defect size and distribution on fatigue life.

This paper investigates the fatigue performance of Q500qENH weathering steel welded joints under low-temperature condition of −40 °C. Fatigue tests were conducted on V-groove butt joints under four stress ranges and cross-shaped welded joint under three stress ranges, to obtain S-N curves with a 95% survival probability. The fracture morphology of the specimens was examined using scanning electron microscopy (SEM) to analyze the fatigue fracture mechanism of the welded joints. Subsequently, based on the linear elastic fracture mechanics (LEFM) approach, numerical simulations of fatigue crack propagation in the weathering steel welded joints at low temperature were performed using ABAQUS 2023 and FRANC3D 8.4.0 software. After validating the model’s effectiveness, a parametric analysis was conducted on key factors influencing fatigue crack growth, including initial crack size, location, and angle, to elucidate their effects on fatigue crack propagation mechanisms.

## 2. Experimental Details

### 2.1. Preparation of Specimens

**Figure 1:**
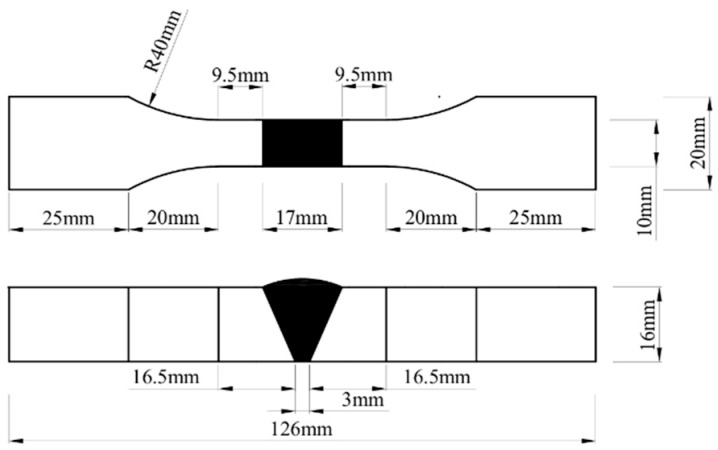
Dimensions of the V-groove butt joint fatigue specimens.

**Figure 2:**
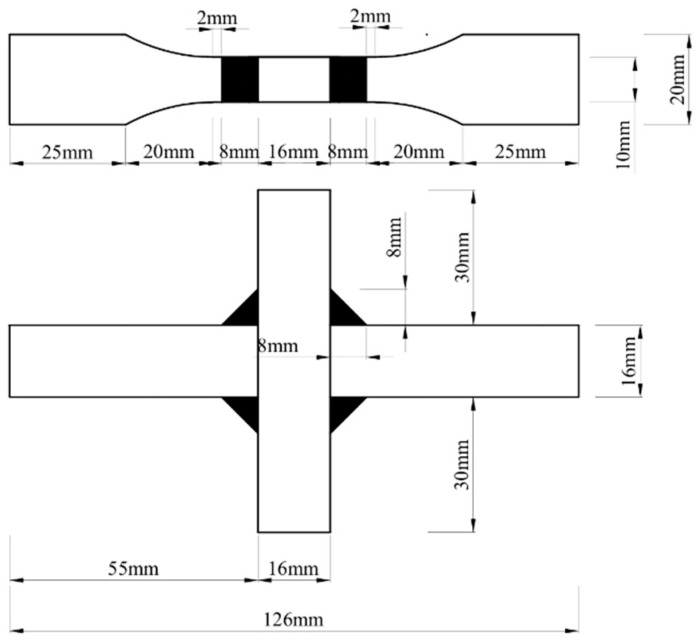
Dimensions of the cross-shaped welded joint fatigue specimens.

Both the V-groove butt joint specimens and the cross-shaped welded joint specimens were designed in accordance with Metallic materials—Fatigue testing—Axial force-controlled method (ISO 1099:2017) [25]. The V-groove butt joint specimens were fabricated with a V-groove weld, featuring a weld width of 10 mm and a groove angle of 50°. The cross-shaped welded joint specimens had a fillet weld leg size of 8 mm and a weld width of 10 mm. The specimen dimensions are illustrated in Figure 1 and Figure 2.

Tensile tests were conducted to determine the mechanical properties of the weathering steel base metal used in this study. The yield strength (*f*_y_), ultimate strength (*f*_u_), elongation in percentage after fracture (*A*), yield strength ratio and Young’s modulus (E) of the base metal are listed in Table 1.

### 2.2. Test Details

**Figure 3:**
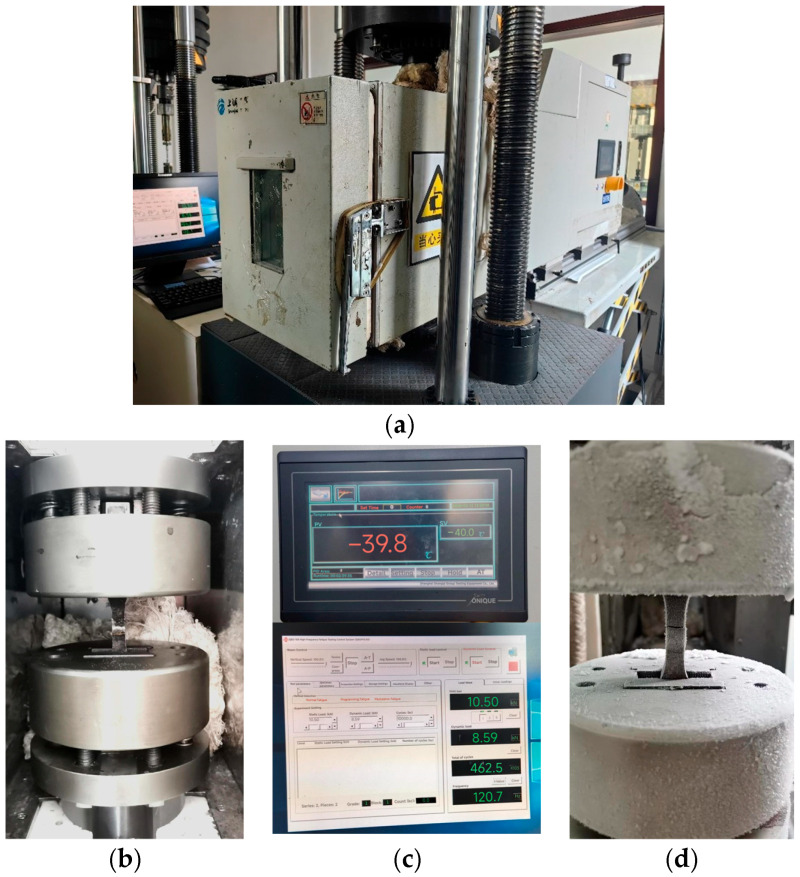
Low-temperature fatigue testing system: (**a**) QDB-100 high-frequency fatigue testing machine; (**b**) specimen mounting configuration; (**c**) digital touchscreen interface for temperature regulation (top), fatigue testing measurement and control system (bottom); (**d**) fractured specimen after low-temperature fatigue testing at −40 °C.

The fatigue tests were conducted at −40 °C using a 20-ton high-frequency fatigue testing machine, as shown in Figure 3, specimens were properly mounted in the testing machine using grips. The constant temperature of the low-temperature environmental chamber was maintained by a cryogenic chamber supplied with liquid nitrogen, with temperature stability (±0.5 °C) monitored throughout testing by platinum resistance thermometers. The loading frequency was 120 Hz and the stress ratio (*R*) of sinusoidal load was 0.1 for all specimens. The applied stress levels for the V-groove butt joints were set at 0.6, 0.5, 0.4, and 0.3 times the yield strength, while those for the cross-shaped welded joints were 0.6, 0.5, and 0.4 times the yield strength. For each stress level, one valid specimen was tested.

To assess temperature fluctuation of specimens during fatigue loading, a preliminary testing was performed on a V-groove butt joint specimen at −40 °C under 348 MPa (0.6*f*_y_) maximum stress. Temperature monitoring confirmed the specimen remained within ±2 °C of the target temperature throughout testing, demonstrating stable thermal conditions under cryogenic fatigue loading.

The formal fatigue test consists of the following steps: specimen clamping, temperature application and stabilization, load application, and specimen fracture. Cyclic loading with a specified stress level and a stress ratio of 0.1 was applied to the specimen until crack initiation and fracture occur, then terminate the test and record the number of fatigue load cycles. The fatigue cycle limit is set to 2 million cycles; if the specimen does not fracture after 2 million cycles, the test is stopped.

## 3. Analysis of Experimental Results

### 3.1. Test Results

Among the seven specimens tested, six experienced fracture failure, and the test results are presented in Table 2. S_max_ represents the maximum stress, calculated according to the prescribed stress levels, MPa; S_min_ represents minimum stress, S_min_ = *R* × S_max_, where *R* = 0.1, MPa; and the stress range (ΔS) is calculated as ΔS = S_max_ − S_min_.

Both joint types show increased fatigue life with lower stress levels. The V-groove butt joints outperform cross-shaped welded joints by 1–2 orders of magnitude in fatigue life under the same stress level. This can be attributed to cross-shaped joints’ higher stress concentrations and defect-prone fillet welds. The V-groove butt joints experience no failure after 2 million cycles at a maximum stress of 174 MPa (0.3*f*_y_), demonstrating a fatigue limit.

The fatigue design section of Eurocode 3 [4] provides the fatigue limits and corresponding S-N curves for various structural details at room temperature. However, existing research on the low-temperature fatigue performance of steel is rarely based on the S-N curve evaluation method and mostly relies on fracture mechanics evaluation methods. Liu [26] and Li [1] investigated the low-temperature fatigue behavior of V-groove butt joints in DH36 shipbuilding steel and cross-shaped welded joints in Q420C high-strength steel, respectively, under −40 °C conditions. This section extracts the fatigue test results to fit its S-N curve for comparison with the experimental results in this study.

**Figure 4:**
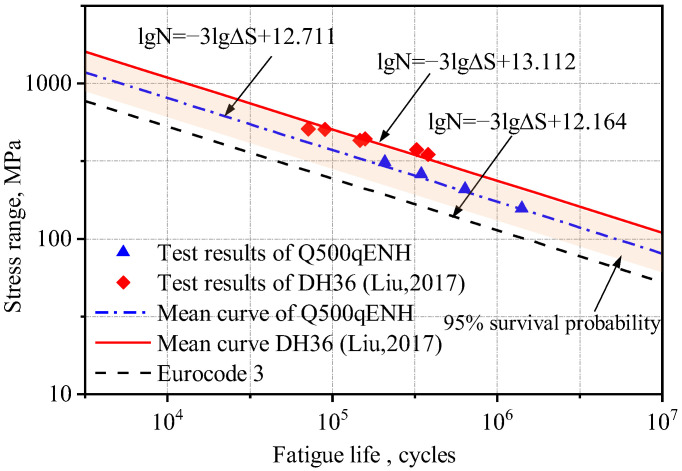
Comparison of S-N curves for the V-groove butt joints with previous studies [4,26].

**Figure 5:**
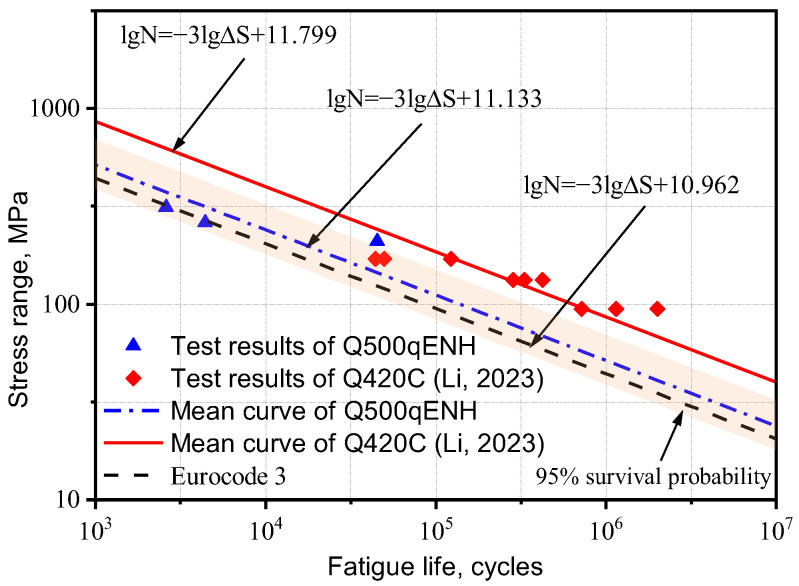
Comparison of S-N curves for the cross-shaped welded joints with previous studies [1,4].

Based on the method proposed by Hobbacher [16], the design S-N curve with a 95% survival probability was derived using a fixed slope of −0.333 [27]. Linear regression was applied to the experimental data, and the low-temperature fatigue test data from this study and references [1,26] were plotted in logarithmic coordinates, along with the corresponding S-N curves from Eurocode 3 for matching structural details, as shown in Figure 4 and Figure 5.

The S-N curves of V-groove butt joints and cross-shaped welded joints at −40 °C obtained from the experimental results in this study lie between those reported in references [1,26] and Eurocode 3. Q500qENH weathering steel shows a longer fatigue life than Eurocode 3 specifications but a shorter one than Liu Zijie and Li Qiyu’s results. Based on a 95% survival probability, the calculated fatigue strengths of the two types of welded joints at −40 °C were 137.0 MPa and 40.7 MPa, respectively, both exceeding the design fatigue strengths of corresponding joints in Eurocode 3, 90.0 MPa and 36 MPa. Furthermore, the specified fatigue strengths in Eurocode 3 represent 66% (V-groove butt joints) and 88% (cross-shaped joints) of Q500q’s values, while DH36 and Q420C steels demonstrate design fatigue strengths that are 136% and 167% of Q500qENH, respectively. This indicates that low temperatures can enhance the design fatigue strength of welded joints to a certain extent. Therefore, Eurocode 3 can be applied to the fatigue design of this batch of welded joint specimens and provides conservative predictions of fatigue life.

### 3.2. Test Observations

#### 3.2.1. Fracture Locations

**Figure 6:**
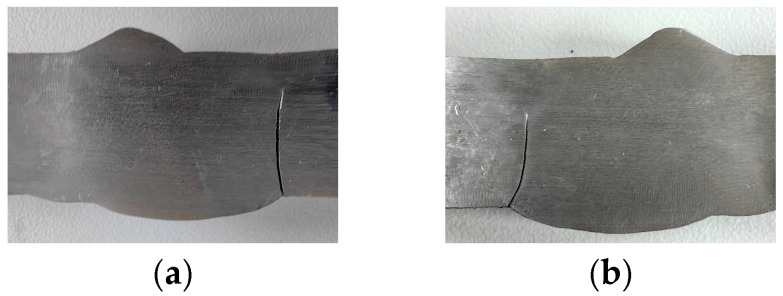
The final state of crack propagation in specimen B-2: (**a**) the front side; (**b**) the back side.

For the V-groove butt joint fatigue specimens, cracks consistently initiated at the weld toe and subsequently propagated along the interface between the weld and base metal, remaining close to the base metal. Figure 6 shows the crack morphology of specimen B-2 after testing. The crack originated at the weld toe and extended through the thickness direction, with the final crack lengths on the two sides measuring 8.96 mm and 12.32 mm, respectively, accounting for 56% and 77% of the total thickness of 16 mm.

**Figure 7:**
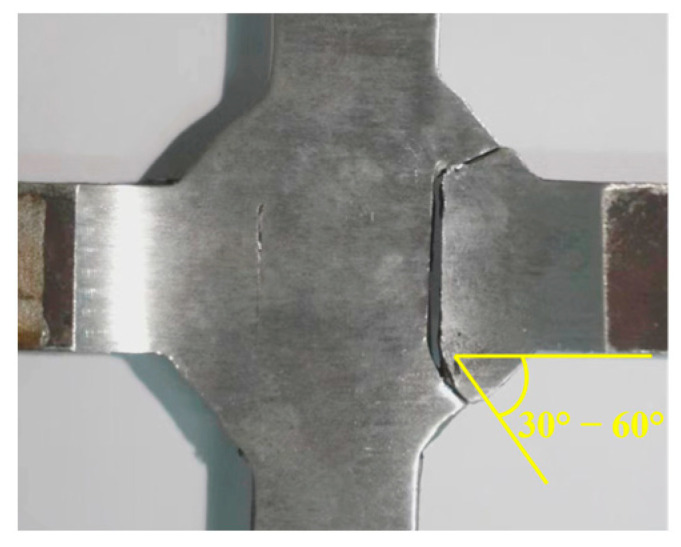
The final state of crack propagation in specimen F-1.

In the cross-shaped welded joint specimens, cracks consistently initiated at the fillet weld and subsequently propagated toward the weld root, ultimately leading to fracture. Figure 7 shows the crack morphology of specimen F-1 after testing. The crack propagation path formed an angle of 30° to 60° with the length direction of the middle plate, and the fracture trajectories on both sides of the weld were asymmetrical.

#### 3.2.2. Macroscopic Fracture Morphology

The macroscopic fracture surface of fatigue specimens primarily consists of three zones: crack origin (region I), crack propagation (region II), and instantaneous fracture (region III). These regions are typically observed and distinguished under low magnification in a scanning electron microscope.

**Figure 8:**
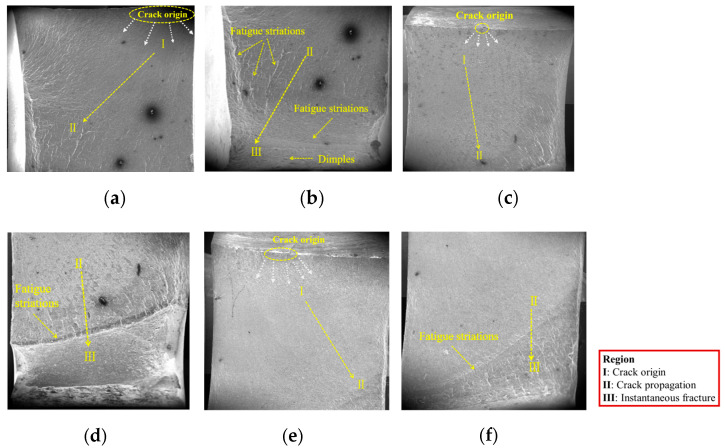
The macroscopic fracture morphologies of the V-groove butt joints: (**a**) Part 1 of B-1; (**b**) Part 2 of B-1; (**c**) Part 1 of B-2; (**d**) Part 2 of B-2; (**e**) Part 1 of B-3; (**f**) Part 2 of B-3.

**Figure 9:**
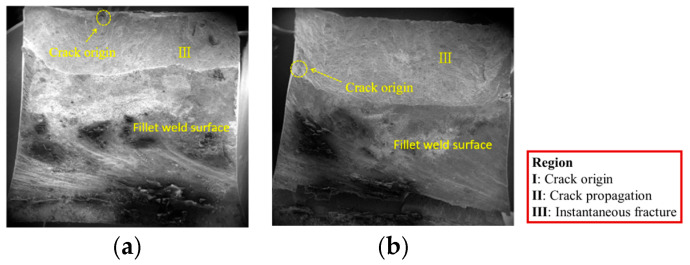
The macroscopic fracture morphologies of the cross-shaped welded joints: (**a**) F-1; (**b**) F-2.

Figure 8 and Figure 9 show the macroscopic fracture morphologies of V-groove butt joints and cross-shaped welded joints, respectively.

The V-groove butt joint specimen exhibits distinct zone demarcation. The fatigue initiation zone presents a relatively smooth interface due to repeated compression and friction during loading. The crack propagation zone contains numerous fatigue striations, which are formed by fatigue crack growth from initiation sites on different planes. Upon entering the rapid fracture zone, a prominent fatigue striation appears, and the rapid fracture zone exhibits a rough surface with abundant dimples.

Defects can be observed inside the fracture surfaces of specimens B-1 and B-2, which may result from the magnification of internal casting defects or welding flaws during loading. Specimen B-3, subjected to lower stress levels with significantly reduced casting defects, displays a more intact fracture surface. As the stress level decreases, the number of fatigue striations in the crack propagation zone gradually diminishes.

The fracture plane of the cross-shaped welded joint forms a certain angle with the plane of the welded plate, allowing simultaneous observation of both the fracture surface and non-fracture surface during fractography. As shown in Figure 9, there is a boundary line between the fracture surface and the non-fracture surface, with the fracture surface above the line and the fillet weld surface below. Macroscopically, it is difficult to clearly distinguish the three typical zones of the fatigue fracture surface. Only the fatigue crack initiation sites can be identified, with both specimens exhibiting crack origins near the edge of the joint.

#### 3.2.3. Microscopic Fracture Morphology

All specimens were sectioned using wire cutting at a distance of 10 mm from the fracture surface and examined via field-emission scanning electron microscopy (FE-SEM) to analyze the microscopic fracture morphology. The following analysis focuses on the microscopic morphology of each macroscopic region of fatigue fracture, exploring the formation mechanism of each region. Since the V-groove butt joint and cross-shaped welded joint exhibit similar microscopic features across the three zones, they are not distinguished in the analysis [28].

**Figure 10:**
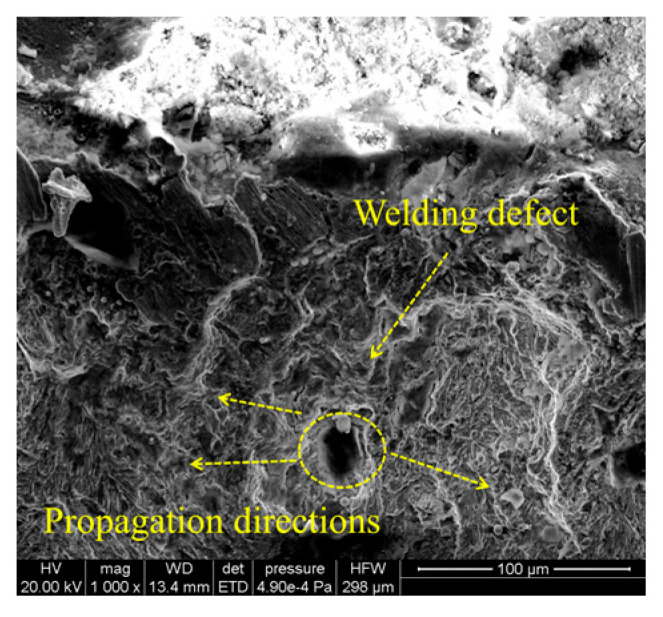
Microscopic fracture morphology of crack initiation zone.

**Figure 11:**
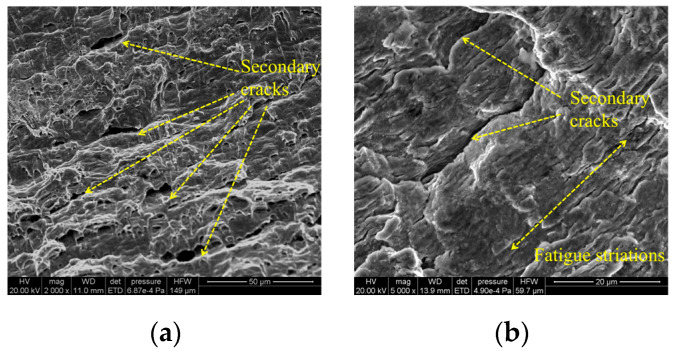
Microscopic fracture morphology of crack propagation zone: (**a**) 2000× magnification; (**b**) 5000× magnification.

**Figure 12:**
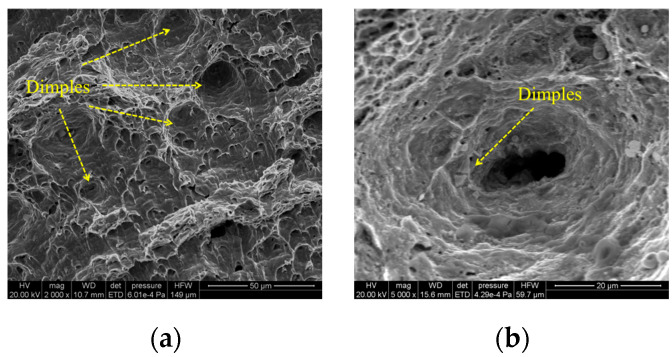
Microscopic fracture morphology of instantaneous fracture zone: (**a**) 2000× magnification; (**b**) 5000× magnification.

1. Crack initiation zone: The fatigue initiation zone (Figure 10) typically originates from welding defects, such as pores, inclusions, or microcracks. Under cyclic loading, stress concentration occurs at these defects, leading to crack nucleation. The initiation site often appears relatively smooth due to repeated compression and rubbing during early crack propagation.
2. Crack propagation zone: The crack propagation zone (Figure 11) exhibits fatigue striations and secondary cracks at different magnification levels. These secondary cracks form due to the fracture of fatigue striations and propagate perpendicular to the main crack growth direction, serving as a key indicator of fatigue crack advancement.
3. Instantaneous fracture zone: The rapid fracture zone (Figure 12) is characterized by dimples, whose formation is independent of stress levels. The orientations of different dimples vary. One type is aligned with the specimen’s fracture direction, referred to as elongated dimples; the other type is perpendicular to the fracture plane, known as equiaxed dimples. Both V-groove butt joints and cross-shaped welded joints exhibit these two types of dimple morphologies.

## 4. Numerical Simulation

### 4.1. Simulation Details

**Figure 13:**
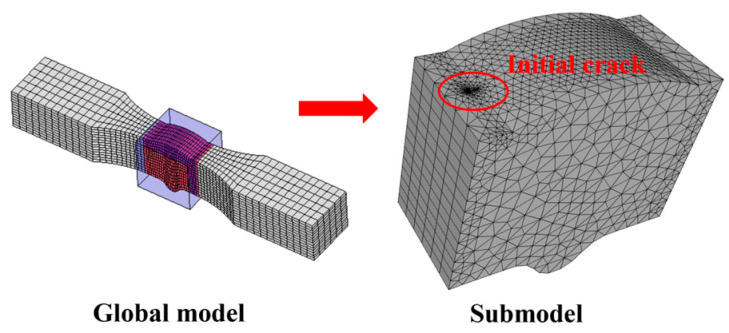
The initial crack inserted in the V-groove butt joints.

**Figure 14:**
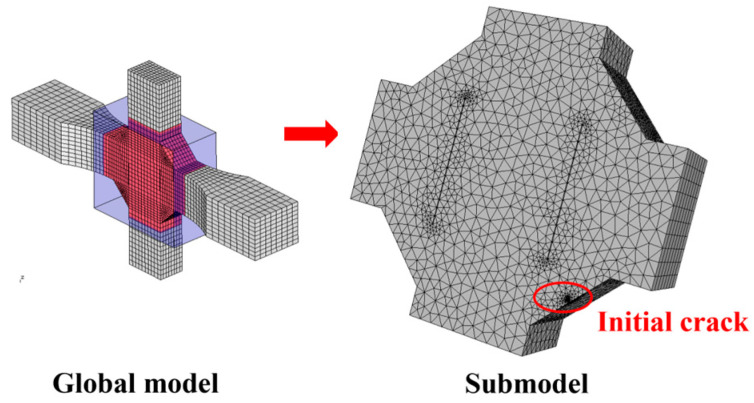
The initial crack inserted in the cross-shaped welded joints.

This section is based on the theory of LEFM and utilizes the FRANC3D fracture analysis software to analyze the fatigue performance of Q500qENH weathering steel welded joints. The analysis involves the following main steps:

1. Finite element model construction: A complete finite element model of the fatigue test specimen is constructed in ABAQUS, and preliminary static stress analysis is performed based on the stress levels corresponding to each specimen in the test. The material parameters adopt the tensile test data of Q500qENH weathering steel at −40 °C, with an elastic modulus E = 2.08 × 10^5^ MPa and a Poisson’s ratio μ = 0.3.
2. Initial crack insertion: An appropriate initial crack is inserted into the model, followed by mesh refinement. Based on phenomena observed in relevant fatigue tests, this study selects elliptical surface cracks as the initial cracks. The size of the initial crack is primarily determined by two parameters: the semi-major axis length (a) and the semi-minor axis length (c) of the elliptical crack. In this section, referencing the research findings of Zong [29], both a and c are set to 0.4 mm. According to static analysis results, the region near the weld toe in the parallel section of the V-groove butt joint specimens exhibits the highest stress. Combined with fatigue test observations, fatigue fractures typically initiate at the specimen edge near the weld toe. Therefore, the initial crack location is selected at the interface near the weld toe, where the initial crack plane is perpendicular to the direction of the external load, and the distance from the weld toe is set to 0.2 mm, as shown in Figure 13. For the cross-shaped welded joint, significant stress concentrations occur at both the weld toe and weld root due to geometric discontinuities, leading to stress concentration under applied loads. Based on the observed fracture locations in the tests, the initial crack is placed at the edge of the fillet weld surface along the width direction, as shown in Figure 14.
3. Stress analysis and crack propagation: Perform stress analysis on the model after inserting the initial crack to obtain the stress redistribution in the model. Calculate the stress intensity factor in FRANC3D, propagate the crack, and remesh the model. After crack propagation, conduct stress analysis again and cycle through the above steps until the crack reaches the critical size. According to experimental results, specimen failure occurs when the crack size reaches 56% to 77% of the thickness. Based on the findings of Arora et al. [30], this study adopts two-thirds of the specimen thickness as the critical crack size.

### 4.2. Simulation Results

#### 4.2.1. Fatigue Life

After crack propagation analysis, the fatigue life of the specimen is calculated according to Equation (1) [31].

(1) $$N=\int_{a_{0}}^{a_{f}}\frac{1}{C{\left(\Delta K_{e}\right)}^{m}}da$$

where *N* is the fatigue life, *a_0_* is the initial crack size, *a_f_* is the critical crack size, and *C* and *m* are fatigue crack growth rate parameters.

Based on the crack growth rate test results of Q500qENH steel at −40 °C, this study adopts *C* = 6.966 × 10^−12^ and *m* = 2.751 to account for the effect of low temperature on crack propagation.

**Figure 15:**
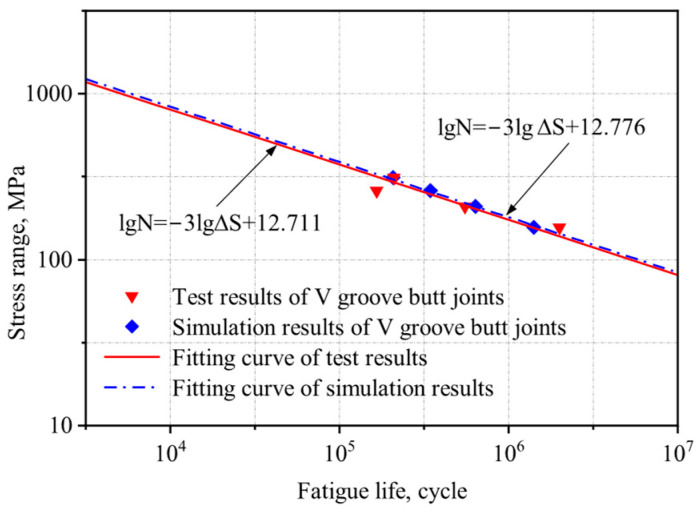
Comparison of experimental and numerical simulation S-N curves for the V-groove butt joints.

**Figure 16:**
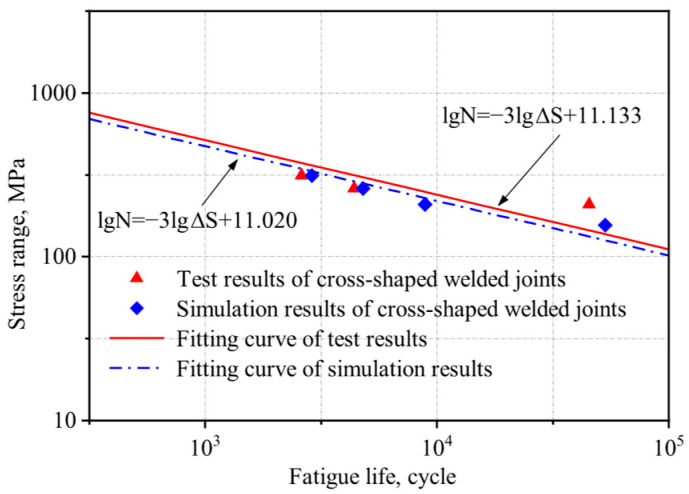
Comparison of experimental and numerical simulation S-N curves for the cross-shaped welded joints.

The stress amplitudes and corresponding fatigue lives of each specimen from both experimental measurements and numerical simulations were plotted on a logarithmic scale, and the S-N curve was fitted accordingly. The resulting curve and its fitting equation are presented in Figure 15 and Figure 16.

There is a certain discrepancy between the numerical simulation results and the experimental data, which may be attributed to the following factors: (a) Idealized simulation conditions: The numerical model does not account for welding defects or casting defects, leading to an overestimation of fatigue life. Moreover, material properties in simulations are typically homogeneous, whereas actual specimens may contain microstructural irregularities. (b) Potential experimental errors: These include dimensional deviations introduced during specimen machining and load application inaccuracies due to the limitations of the testing apparatus.

Under the same stress ratio, the stress amplitude and fatigue life exhibit an approximately linear relationship in double logarithmic coordinates. In logarithmic coordinates, the error between the experimentally and simulated fatigue life results is less than 5%, demonstrating good agreement between the two datasets. This consistency validates the reliability of the FEM for fatigue life prediction.

#### 4.2.2. Crack Propagation Morphology

**Figure 17:**
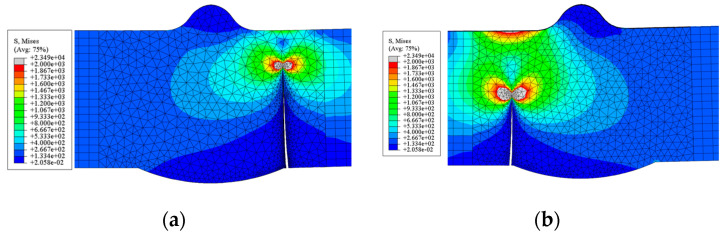
The simulated final crack propagation states of the V-groove butt joints: (**a**) front side of the specimen; (**b**) back side of the specimen.

**Figure 18:**
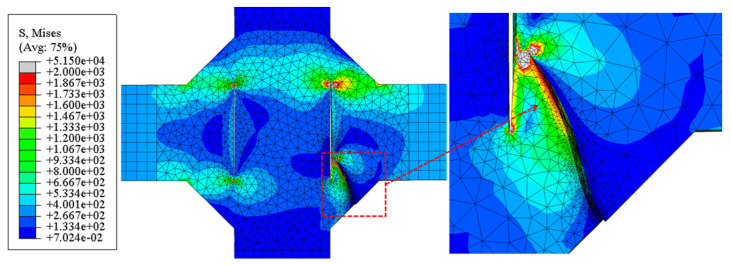
The simulated final crack propagation states of the cross-shaped welded joints.

The simulated final crack propagation states of the V-groove butt joint and cross-shaped welded joint are presented in Figure 17 and Figure 18. By compared with the experimentally observed crack propagation morphology (Figure 6 and Figure 7), it can be seen that in the V-groove butt joint tests, the crack lengths on both sides of the specimen thickness direction exhibited asymmetry. The simulation results also captured this phenomenon, indicating that the simulation method of inserting initial cracks at the width–thickness interface can effectively simulate the initial defects in the specimen.

For the cross-shaped welded joint, fracture occurred at the fillet welds on both sides, forming an angle of 30° to 60° relative to the external tensile direction. Figure 18 shows that under cyclic loading, the initial crack propagated from the fillet weld edge toward the central weld gap until structural failure.

In terms of the final failure state, the numerical simulations and experimental results exhibited consistent failure modes. The agreement in crack propagation morphology and crack growth process between tests and simulations confirms the reliability of the numerical approach.

In conclusion, by employing ABAQUS for geometric modeling, introducing an initial crack of 0.4 mm, and integrating FRANC3D for 3D crack propagation analysis, the crack propagation of weathering steel V-groove butt joints and cross-shaped welded joints can be accurately predicted.

## 5. Parameter Study

This study found that the size, location, and angle of initial cracks are key factors affecting the fatigue life of welded joints [32,33]. This section focuses on the V-groove butt joints and cross-shaped welded joints of Q500qENH weathering steel and presents typical numerical examples to investigate the influence patterns of these parameters.

### 5.1. Initial Crack Size

**Figure 19:**
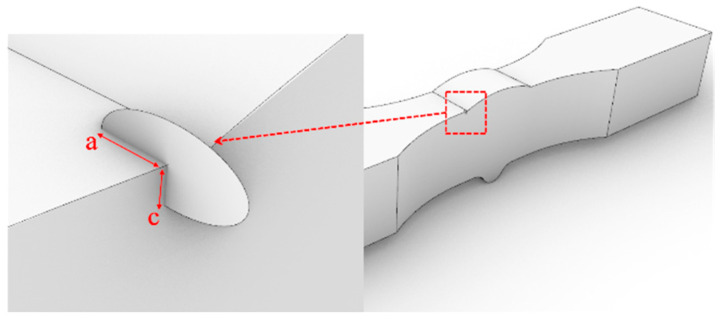
The initial crack of the V-groove butt joints.

**Figure 20:**
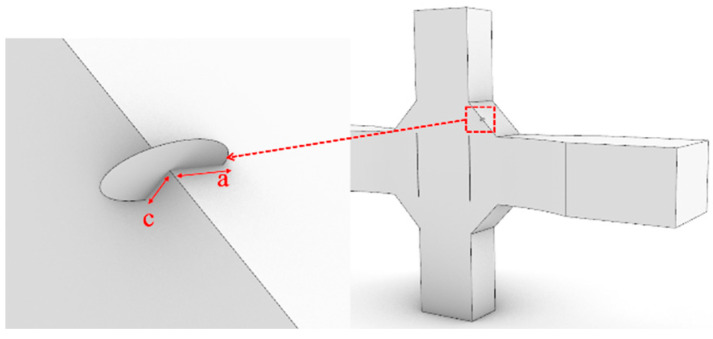
The initial crack of the cross-shaped welded joints.

To investigate the influence of initial crack size on the fatigue performance of the two welded joints, seven numerical cases were designed for each joint (*a/c* = 1, 2, 3, 4, 1/2, 1/3, 1/4). The initial crack location is as shown in Figure 19 and Figure 20.

**Figure 21:**
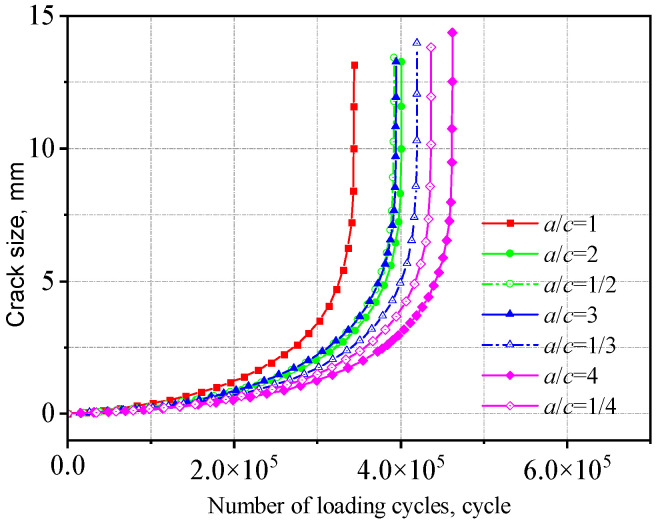
Influence of initial crack size on the V-groove butt joints.

**Figure 22:**
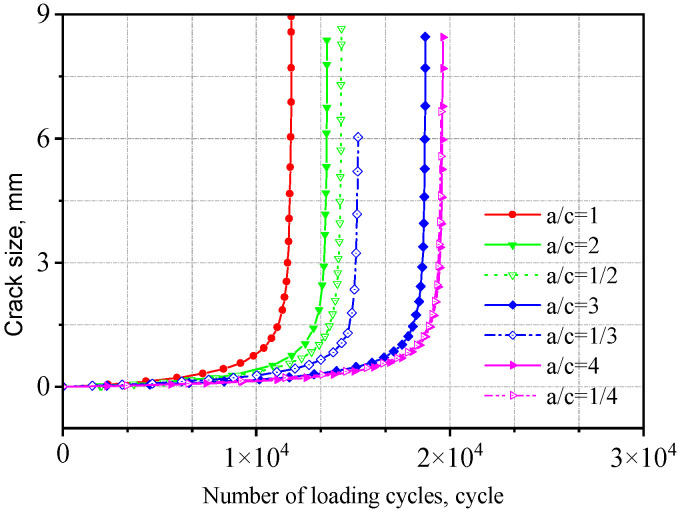
Influence of initial crack size on the cross-shaped welded joints.

The fatigue life obtained from crack propagation analysis is presented in Table 3. The a-N curves for two types of welded joints with initial crack aspect ratios (*a/c*) ranging from 4 to 1/4 are shown in Figure 21 and Figure 22.

As *a/c* increases, the fatigue life initially decreases and then increases. That is, under the same cyclic stress, a smaller difference between the major and minor semi-axes leads to a larger crack extension length, which has a more detrimental effect on crack propagation. This is because when the major and minor semi-axes are equal (*a/c* = 1), the initial crack has a larger surface area, resulting in a more extensive region adversely affecting fatigue crack growth and consequently inducing a broader stress concentration zone, accelerating crack propagation. Compared to the V-groove butt joints, the fatigue life of the cross-shaped welded joints is more sensitive to changes in initial crack size. The fatigue life when *a*/*c* = 1 is approximately 50% of that when *a/c* = 4.

The initial crack size has a relatively limited effect on the final stress distribution at the crack propagation stage for both types of welded joints, except the larger the difference between the major and minor semi-axes (a/c), the greater the stress at the crack tip.

**Figure 23:**
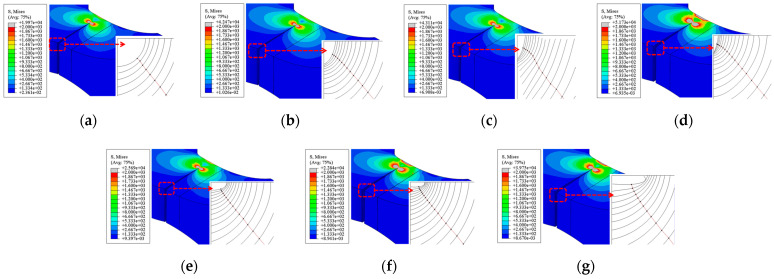
Crack propagation path and final stress distribution in the V-groove butt joints: (**a**) *a/c* = 1; (**b**) *a/c* = 2; (**c**) *a/c* = 3; (**d**) *a/c* = 4; (**e**) *a/c* = 1/2; (**f**) *a/c* = 1/3; (**g**) *a/c* = 1/4.

Figure 23 shows the crack propagation trajectory and final stress contour of the V-groove butt joint. When a/c = 1, the incremental crack extension per step is nearly uniform along the crack front in the early stages. However, when a/c ≠ 1, the early-stage crack extension varies significantly along the crack front, but this difference gradually diminishes in later stages, eventually converging to a state similar to that of a/c = 1.

### 5.2. Initial Crack Location

**Figure 24:**
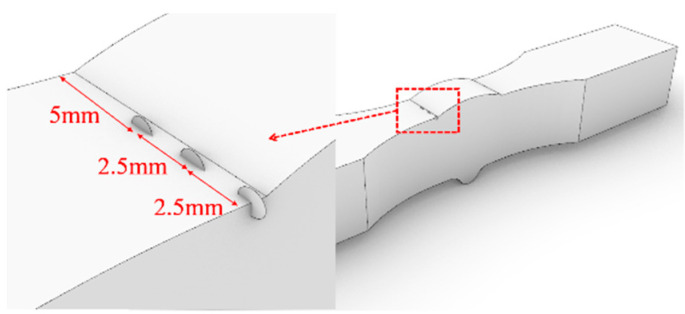
Initial crack locations of the V-groove butt joints.

As shown in Figure 24, three locations along the width direction near the weld toe of the V-groove butt joints were selected for fatigue crack growth analysis: the edge, 1/4 width and 1/2 width.

**Figure 25:**
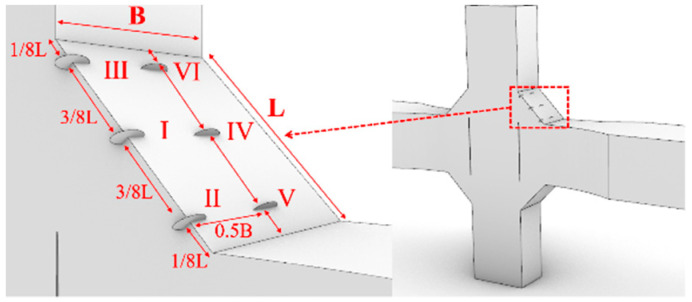
Initial crack locations of the cross-shaped welded joints.

For the cross-shaped welded joints, the initial crack was located on the surface of the fillet weld. The width and length of the weld surface were denoted as B and L, respectively. Six different positions (labeled I to VI) were chosen for inserting the initial crack, as illustrated in Figure 25.

**Figure 26:**
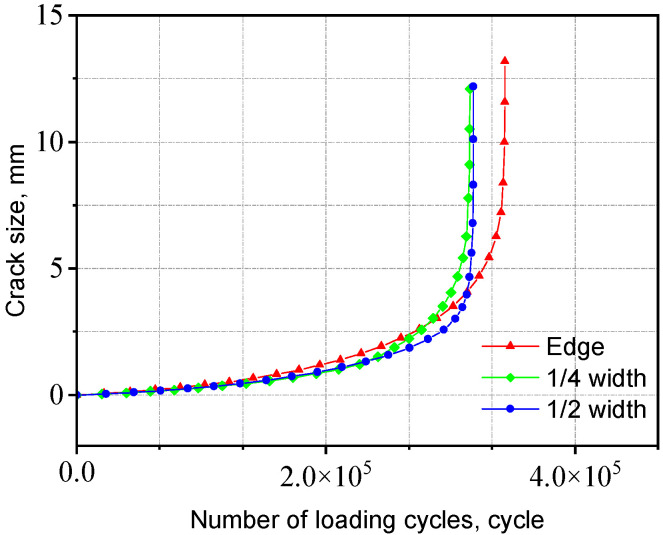
Influence of initial crack location on the V-groove butt joints.

The a-N curves for V-groove butt joints with different initial crack locations are shown in Figure 26. The results indicate that the initial crack position has a relatively minor influence on the fatigue life of the joint. This can be attributed to the uniform stress distribution near the weld toe, where the stress concentration levels at different initial crack locations are similar.

**Figure 27:**
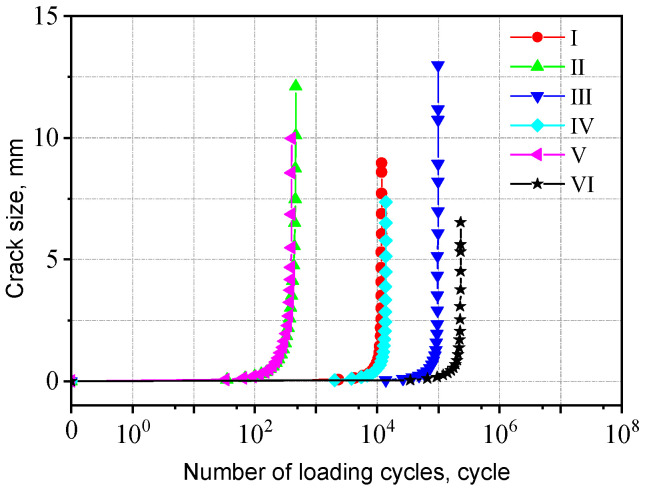
Influence of initial crack location on the cross-shaped welded joints.

The a-N curves for cross-shaped welded joints with different initial crack locations are shown in Figure 27. The fatigue life differences are negligible when initial cracks are inserted at varying positions along the width of the fillet weld surface. In contrast, inserting initial cracks at different positions along the length direction of the fillet weld surface results in significant variations in fatigue life—the fatigue life at Position II is only 1/200 of that at Position III. The corresponding fatigue lives for initial cracks inserted in each region are listed in Table 4. When the initial crack is located near the weld toe of the loaded plate, the fatigue life is shorter, whereas it is longer when near the weld toe of the non-loaded plate. This is because the stress near the weld toe of the loaded plate is higher compared to the region near the non-loaded plate. Consequently, initial cracks in this area induce greater stress concentration, accelerating crack propagation and reducing fatigue life. The crack propagation behavior for both joint types exhibited distinct patterns influenced by initial crack locations, with crack propagation path and stress distributions analyses provided in Appendix A.

### 5.3. Initial Crack Angle

**Figure 28:**
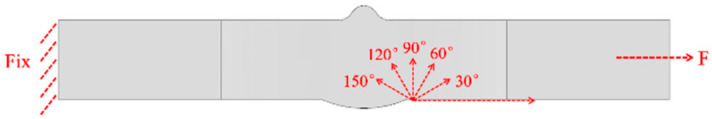
Initial crack angles of the V-groove butt joints.

**Figure 29:**
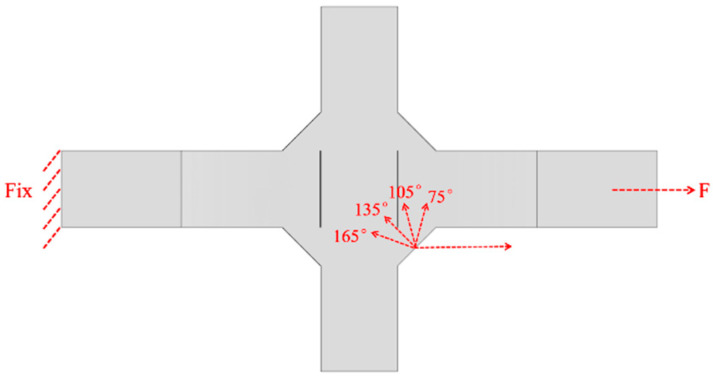
Initial crack angles of the cross-shaped welded joints.

The angle between the initial crack and the direction of external loading is defined as the initial crack angle. As shown in Figure 28, for the V-groove butt joints, initial crack angles of 30°, 60°, 90°, 120°, and 150° were selected, with the initial cracks set at the weld toe and the edge along the weld width direction. As shown in Figure 29, for the cross-shaped welded joints, with the weld plane as the reference surface and considering that the angle between the fillet weld plane and the external loading direction is 45°, initial crack angles of 75°, 105°, 135°, and 165° were chosen. The initial cracks were all located at the midpoint of the edge line along the fillet weld width direction.

**Figure 30:**
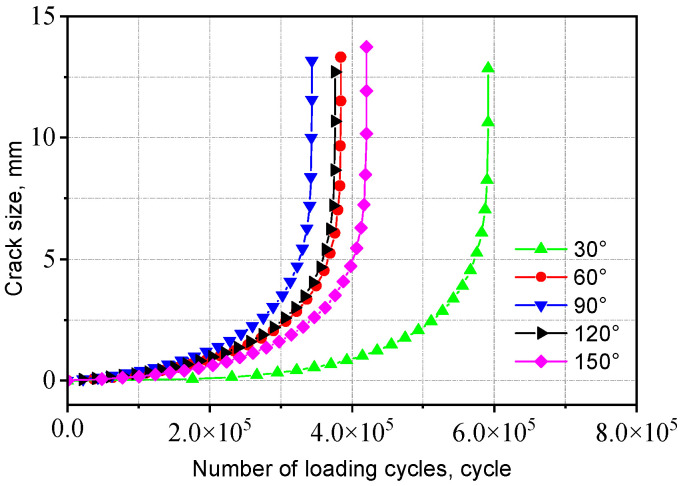
Influence of initial crack angle on the V-groove butt joints.

The a-N curves for the V-groove butt joints with different initial crack angles are shown in Figure 30. When the initial crack angle is 90°, the fatigue life is the smallest, as the crack plane deviates further from this angle, the corresponding fatigue life increases. This is because a larger initial crack angle results in a smaller component of the external tensile force perpendicular to the crack, leading to a lower average stress in the crack vicinity. Additionally, the stress distribution near the weld toe is more complex compared to the other side. Consequently, when the initial crack angle is 30°, the corresponding fatigue life reaches its maximum value, whereas at an included angle of 90°, the fatigue life is approximately 60% of that at 30°.

**Figure 31:**
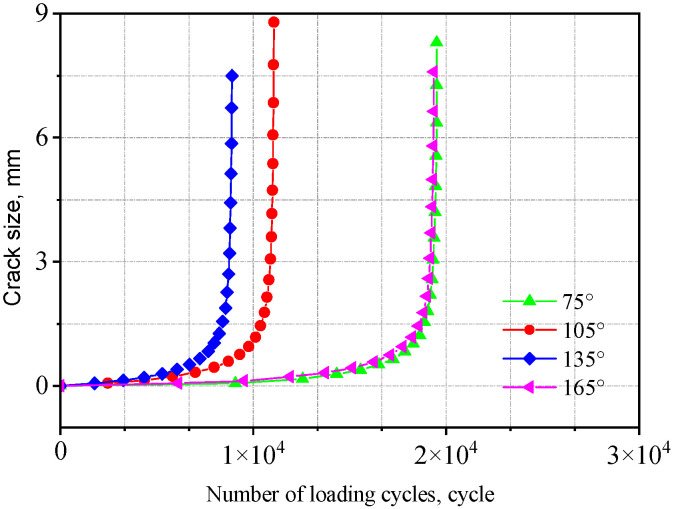
Influence of initial crack angle on the cross-shaped welded joints.

The a-N curves for the cross-shaped welded joints with different initial crack angles are shown in Figure 31. As can be seen from the figure, the fatigue life is the smallest when the initial crack angle is 135°; the fatigue life reaches its maximum at initial crack angles of 75° and 165°. In other words, when the initial crack plane is simultaneously perpendicular to both the surface and the side of the fillet weld, the fatigue life is minimized. As the crack plane deviates further from this orientation, the corresponding fatigue life increases. Specifically, the fatigue life at an initial crack angle of 135° is approximately 50% of that at an initial crack angle of 75°.

## 6. Conclusions

This paper investigates the fatigue performance of Q500qENH weathering steel welded joints at −40 °C low temperature. Low-temperature fatigue tests were conducted on two types of welded joint specimens, and the fracture morphology was examined through scanning electron microscopy (SEM). Subsequently, numerical simulations of fatigue crack propagation in the weathering steel welded joints under low-temperature conditions were performed, along with a parametric analysis of key factors influencing fatigue crack growth. The main conclusions are as follows:

1. The S-N curves with 95% reliability were established for both types of welded joints. A comparison with the corresponding S-N design curves for structural details in Eurocode 3 revealed that low temperature moderately enhances the design fatigue strength of both welded joints. Eurocode 3 can be applied for fatigue design of this batch of welded joint specimens and provides a relatively conservative fatigue life prediction.
2. Fatigue cracks in the specimens typically initiated from welding defects. In all the fractured V-groove butt joint specimens, cracks originated at the weld toe and propagated near the fusion line, predominantly within the base metal. All the cross-shaped welded joint specimens initiated cracks at the fillet weld, followed by propagation toward the weld root, ultimately leading to fillet weld fracture.
3. Initial crack size, angle, and location all influence the fatigue life and crack propagation path of both welded joints to some extent. The fatigue life of cross-shaped welded joints is most sensitive to initial crack location—when the crack initiates near the weld toe of the load-bearing plate, the fatigue life is only 1/200 of that when the crack initiates near the weld toe of the non-load-bearing plate.

## Figures and Tables

**Table 1:** Mechanical properties of Q500qENH weathering steel base material.

Content	*f*_y_ (MPa)	*f*_u_ (MPa)	*A* (%)	*f*_y_/f_u_	*E* (GPa)
Value	580.07	683.48	18.18	0.85	207.61

**Table 2:** Fatigue test results.

No. of Specimen	Stress Level	S_max_ (MPa)	S_min_ (MPa)	Stress Range ΔS (MPa)	Fatigue Life N (Cycle)
B-1	0.6*f*_y_	348	34.8	313.2	210,700
B-2	0.5*f*_y_	290	29.0	261.0	165,900
B-3	0.4*f*_y_	232	23.2	208.8	553,100
B-4	0.3*f*_y_	174	17.4	156.6	2,000,000
F-1	0.6*f*_y_	348	34.8	313.2	2600
F-2	0.5*f*_y_	290	29.0	261.0	4400
F-3	0.4*f*_y_	232	23.2	208.8	45,300

**Table 3:** Fatigue life of two types of welded joints with different aspect ratios.

Semi-Major Axis (mm)	Aspect Ratio	Fatigue Life of the V-Groove Butt Joints (Cycle)	Fatigue Life of the Cross-Shaped Welded Joints (Cycle)
0.40	1	343,762	11,818
0.40	2	401,159	13,662
0.40	3	394,507	18,733
0.40	4	462,002	19,642
0.20	1/2	392,042	14,394
0.13	1/3	419,798	15,249
0.10	1/4	436,702	19,535

**Table 4:** Fatigue life of the cross-shaped welded joints with different initial crack location.

Position	I	II	III	IV	V	VI
Fatigue life (cycle)	11,818	465	98,071	13,725	399	231,387

**Figure A1:**
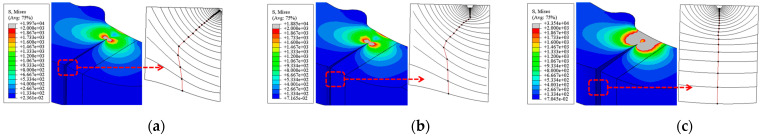
Crack propagation path and final stress distribution in the V-groove butt joints: (**a**) edge; (**b**) 1/4 width; (**c**) 1/2 width.

**Figure A2:**
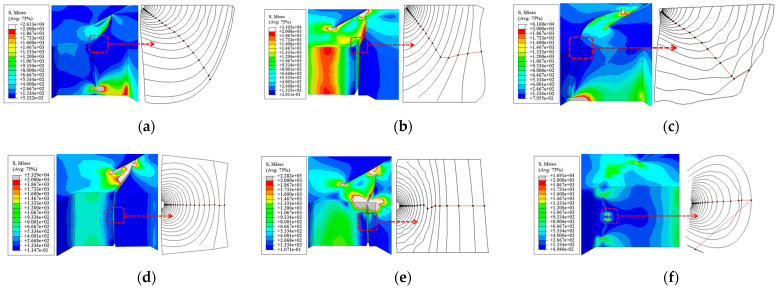
Crack propagation path and final stress distribution in the cross-shaped welded joints: (**a**) Position I; (**b**) Position II; (**c**) Position III; (**d**) Position IV; (**e**) Position V; (**f**) Position VI.

## Data Availability

The original contributions presented in this study are included in the article. Further inquiries can be directed to the corresponding authors.

## Appendix A

Figure A1 shows the crack propagation paths and final stress distributions at three different locations of the V-groove butt joint. As observed, the final stress fields near the crack tips at all three locations exhibit a “heart-shaped” distribution, with stresses gradually decreasing outward from the tip, while the remaining regions remain in the elastic stage. Regarding crack propagation paths, due to different initiation positions, the crack at the width edge propagates simultaneously along both the width and thickness directions until traversing the entire surface and the cracks at the 1/4-width and 1/2-width positions initially extend along the width direction before ultimately spanning the entire surface.

Figure A2 presents the crack propagation paths and final stress contours for the cross-shaped welded joint under various initial crack locations. Cracks at positions I, II, and III all initiate from the interface region. Among them, cracks at positions II and III fully penetrate the fillet weld width, with all three cracks propagating toward the weld gap. Cracks at positions IV, V, and VI initiate from the fillet weld surface, gradually penetrating the weld width while extending along the thickness direction. However, the crack at position Ⅵ, being farther from the weld gap, develops more slowly—at failure, it penetrates the internal width of the weld but has not yet traversed the surface width of the weld.
